# Downregulation of long non-coding RNA PVT1 enhances fracture healing via regulating microRNA-497-5p/HMGA2 axis

**DOI:** 10.1080/21655979.2021.1987099

**Published:** 2021-10-20

**Authors:** Xiang Ji, Zhiqing Li, Wei Wang, Jun Chen

**Affiliations:** a Department of Hand and Foot Trauma Surgery, Qingdao Central Hospital, Qingdao, China; b Department of Emergency Surgery, Qingdao Central Hospital, Qingdao, China; c Department of Second Oncology Radiotherapy, Qingdao Central Hospital, Qingdao, China

**Keywords:** Fragility fracture, LncRNA pvt1, MiR-497-5p, HFOB1.19

## Abstract

Fragility fracture is a common and serious complication of osteoporosis. Abnormal expression of long non-coding RNAs is closely related to orthopedic diseases and bone metabolism. In the study, the role of lncRNA PVT1 during fracture healing, and the potential mechanism were explained. In the present study, 80 cases with fragility fracture were collected, serum samples were also collected at 7, 14, 21 days after standardized fixation therapy. qRT-PCR was applied for the measurement of mRNA levels. hFOB1.19 cells were recruited for the cell experiments, and the cell viability and apoptosis were detected. Luciferase reporter gene assay was performed for target gene confirmation. It was found that the level of PVT1 increased gradually, while miR-497-5p showed a downward trend over time in both intra-articular and hand fracture patients, and the changes reached a significant level at 21 day after treatment. In vitro experiments demonstrated that PVT1 knockdown promoted cell proliferation and inhibited cell apoptosis in HFOB1.19 cells. LncRNA PVT1 acts as a competing endogenous RNA (ceRNA) of miR-497-5p, and the influence of PVT1 knockdown on HFOB1.19 cell proliferation and apoptosis was reversed by miR-497-5p inhibition. HMGA2 is the target gene of miR-497-5p. It was concluded that LncRNA PVT1 silencing may enhance fracture healing via mediating miR-497-5p/HMGA2 axis.

## Introduction

Fragility fracture is defined as fracture injury caused by a fall at or below the center of gravity of the body. Fragility fracture is commonly occurred in older people [1]. Fragility fractures are associated with significant morbidity, mortality and disability and are major public health problems worldwide [2]. The number of new fractures in 2010 in the European Union was estimated at 3.5 million. And the number of deaths related to fractures was estimated at 43,000 [3]. The prevalence of osteoporosis is 10.75% in postmenopausal women and 4.29% in men over 50 years old in China [4]. Worse, the incidence rate of hip fracture has already risen by more than 2- to 3-fold in most Asian countries [5]. The recovery process of fragility fracture is slow, and requires a variety of growth factors and other cytokines to cooperate with each other [6]. Although many strategies have been developed to improve the management of fragility fractures and speed up bone regeneration, there are many deficiencies in the treatment options [7]. Therefore, effective bone regeneration for clinical needs is urgently required.

Long non-coding RNAs (LncRNAs) are non-protein coding transcripts longer than 200 nucleotides. As regulatory RNAs, lncRNAs can act as competing endogenous RNAs (ceRNAs) to regulate the expression of downstream target genes [8]. Numerous studies have shown that lncRNAs are involved in bone formation [9]. Multiple lncRNAs have been reported to play an important role in the generation and transformation of osteoblasts and osteoclasts [9,10]. Recent studies have found that the expression level of lncRNA plasmacytoma variant translocation 1 (PVT1) in chondrocytes of knee osteoarthritis is three times more than that of normal chondrocytes [11], and lncRNA PVT1 can regulate the apoptosis of chondrocytes of osteoarthritis through the adsorption of miR-488-3p, thus affecting the occurrence and development of osteoarthritis [12]. However, its role in fracture healing has not been examined.

MicroRNAs (miRNAs) are short endogenous RNAs with the length of about 20 (18–25) bases, and belong to a type of non-coding RNAs. It can regulate the expression of target genes in multicellular organisms by affecting the stability of mRNA and its translation process [13]. Numerous studies have shown that microRNAs are involved in bone formation [14–16]. During the fracture healing, several miRNAs have been identified to be abnormally expressed, and participate in the process of fracture healing through regulating the proliferation and differentiation of osteoblasts [17,18]. MiR-497-5p has been suggested to participate in bone metabolism and is associated with progressive bone loss due to osteoporosis [19]. In osteoporosis patients, miR-497-5p is poorly expressed, and during HFOB1.19 cell differentiation, the expression of miR-497-5p is decreased [20]. Notably, miR-497-5p regulates several disease progression acting as the target gene of lncRNA PVT1 [21]. Therefore, the role of lncRNA PVT1 and miR-497-5p in fracture healing has aroused our interest.

In this study, a total of 80 patients with fragility fracture were enrolled and the dynamic changes of serum PVT1 and miR-497-5p levels after fracture were recorded. The effects of PVT1 and miR-497-5p on the proliferation and apoptosis of osteoblasts were confirmed by gain and loss function experiments. In addition, the synergistic regulation of PVT1 and miR-497-5p and the possible mechanism of action were also explained.

## Materials and methods

### Collection of patients’ plasma samples

80 experimental subjects who underwent bone fracture were selected who were admitted to Qingdao Central Hospital from March 2019 to October 2020. In which, 40 cases had intra-articular fracture (in which 29 cases were vertebrae fracture and 11 cases were hip fracture), including 31 females and 9 males, with the mean age of 69.73 ± 8.62 years old. Another 40 cases had hand fractures, consisting of 27 females and 13 males, the average age was 71.46 ± 9.18. All 40 hand fracture cases were demonstrated to be extra-articular fractures of the distal radius through radiograph. The included criteria were as follows: (1) were all diagnosed with fragility fracture; (2) surgical treatment was the main treatment; (3) are mentally healthy and can communicate normally. The exclusion criteria were as follows: (1) combined with diseases affecting bone metabolism; (2) patients with heart failure, kidney failure, or autoimmune disease; (3) other causes of fracture, such as pathology fracture, open fracture, etc. Plasma samples were collected from all patients within 24 hours after trauma. The blood samples were centrifuged at 1,000 x g for 15 min at 2–8°C, and aliquots of the serum obtained were subsequently stored at −80°C until analysis. In addition, serum samples were also collected from the same patient at 7, 14, 21 days after standardized fixation therapy. Another 40 healthy people who underwent physical examination during the same period were collected as the control group. In the control group, there were 30 females and 10 males, with the mean age of 70.58 ± 5.61) years old. This study was conducted after approval by the ethics committee of Qingdao Central Hospital (ethical register number: 2019–063-05). All the experimental procedures were in accordance with the regulations issued by the ethics committee. All the patients participated in this study voluntarily.

### Cell culture

Human normal osteoblast hFOB1.19 cells were obtained from the American Type Culture Collection (ATCC; Manassas, VA). hFOB1.19 cells were cultured in DMEM medium containing 10% fetal bovine serum and 100 U/ mL antibiotics (penicillin and streptomycin). The culture condition was 37°C, and 5% CO_2_. The cells were placed under an inverted optical microscope (NiKon Co., Japan), when the confluence of the cells reached 70% ~ 80%, trypsin was used for digestion and passage.

### Cell transfection

The sequences of PVT1 were synthesized and cloned into pcDNA3.1 plasmid to construct the overexpression vector of PVT1 (pcDNA3.1-PVT1) by Beyotime Company (Shanghai, China). Sequences of small interfering RNA of PVT (si-PVT1), si-NC, miR-497-5p mimic or inhibitor, mimic-NC, or inhibitor NC were synthesized and provided by Beyotime Company (Shanghai, China). Cells were seeded in six-well plates and allowed to adhere for 24 h. Then the cell transfection was performed using Lipofectamine 2000 (Invitrogen, Waltham, MA, USA), and the experimental operation was carried out according to the instructions.

### RNA extraction and qRT-PCR

Total RNA was extracted by Trizol method, and the concentration was determined by Nanodrop 2000 spectrophotometer (Thermo Scientific). For RNA isolation, 250 μL of serum was homogenized in 750 μL of Trizol (Invitrogen). Then 200 μL of chloroform was added to the sample and the mixed solution was centrifugated. After an additional chloroform extraction and precipitation with isopropanol, the pellet was washed with 70% ethanol. According to the instructions of the reverse transcription kit, 500ng RNA was reverse transcribed to synthesize complementary DNA (cDNA) using Takara reverse transcription kit (Takara, Dalian, China), and the conditions were 37°C for 60 min and 95°C for 5 min. The cDNA was used as a template for quantitative reverse transcription-polymerase chain reaction (qRT-PCR) reaction. The reaction conditions were 95°C for 5 min, 95°C for 15 S, 60°C for 30 S, 72°C for 30 S, with a total of 40 cycles. . For real-time PCR, 1.33 mL diluted RT products were mixed with 10 mL of 26 Taqman PCR master mixture (No AmpErase UNG), 1 mL TaqMan MicroRNA Assay and 7.67 mL Nuclease free water in a final volume of 20 mL according to manufacturer instructions. With GAPDH as internal reference for PVT1 and U6 as internal reference for miR-497-5p, the relative expression levels of lncRNA PVT1 and miR-497-5p were calculated by the 2^−ΔΔCt^ method [22]. The primer sequences were as follows: PVT1 forward, 5ʹ-GCCCCTTCTATGGGAATCACTA-3ʹ and reverse: 5ʹ-GGGGCAGAGATGAAATCGTAAT-3ʹ; GAPDH forward, 5ʹ-CGCTCTCTGCTCCTCCTGTTC-3ʹ and reverse, 5ʹ-ATCCGTTGACTCCGACCTTCAC-3ʹ; miR-497-5p forward, 5ʹ-CCTTCAGCAGCACACTGTGG-3ʹ and reverse, 5ʹ-CAGTGCAGGGTCCGAGGTAT-3ʹ; U6 forward, 5ʹ-CTCGCTTCGGCAGCACA-3ʹ and reverse, 5ʹ-AACGCTTCACGAATTTGCGT-3ʹ;

### Cell proliferation detection

Cells in exponential growth period (the cell density reached 75%) were inoculated into 96-well culture plates with the inoculation density of 2 × 10^3^ and cultured routinely. After cultured for 24, 48, 72 and 96 h, the cells in each group were added with cell counting kit-8 (CCK-8) solution (Beyotime Company of Biotechnology, Shanghai, China) of 10 μL, and incubated for 10 min. The absorbance at 450 nm was measured by enzyme plate analyzer.

### Cell apoptosis detection

Cell in the logarithmic phase (the cell density reached 75%) was collected in each group, washed by PBS with pre-cooling. 5 μL Annexin V- fluorescein isothiocyanate (FITC) and 5 μL propidium iodide (PI, Joincare Medicine Company, Zhuhai, Guangdong, China) were added into each well and thoroughly mixed. The cells were incubated at room temperature in the dark for 10 min, and cell apoptosis in each group was detected by Facs Calibur flow cytometry and Cellauest software. Followed by FCM (BD FACS Calibur, BD, USA) using the FL1 channel for Annexin V-FITC and the FL2 channel of PI. Both early apoptotic (Annexin V+/PI-) and late apoptotic (Annexin V+/PI+) cells are included in the cell assay of apoptosis.

### Luciferase reporter gene assay

The binding sites between PVT1 or HMGA2 and miR-497-5p were predicted by the target gene prediction software Starbase and TargetScan. The binding site was mutated by gene mutation technology, and the binding site or mutation site was inserted into luciferase reporter gene vector to construct wild-type vector (WT) or mutant vector (MUT), respectively. The reporter and miR-497-5p mimic or inhibitor were co-transfected into hFOB1.19 cells. After 48 hours of culture, the relative luciferase activity of cells in each group was detected.

### Statistics process

GraphPad 7.0 software was used for statistical analysis of all data, which was represented as mean and standard deviation (SD). Data were checked for normality via the Kolmogorov–Smirnov (K-S) normality test. T test was used between two groups comparison, and one-way analysis of variance (ANOVA) was used among multiple groups. *P* < 0.05 was considered statistically significant.

## Results

In this study, a total of 80 patients with fragility fracture were enrolled and the dynamic changes of serum PVT1 and miR-497-5p levels after fracture were recorded. The effects of PVT1 and miR-497-5p on the proliferation and apoptosis of osteoblasts were confirmed by gain and loss function experiments. In addition, the synergistic regulation of PVT1 and miR-497-5p and the possible mechanism of action were also explained.

### Expression level of PVT1 and miR-497-5p in serum of fracture patients

**Figure 1.**
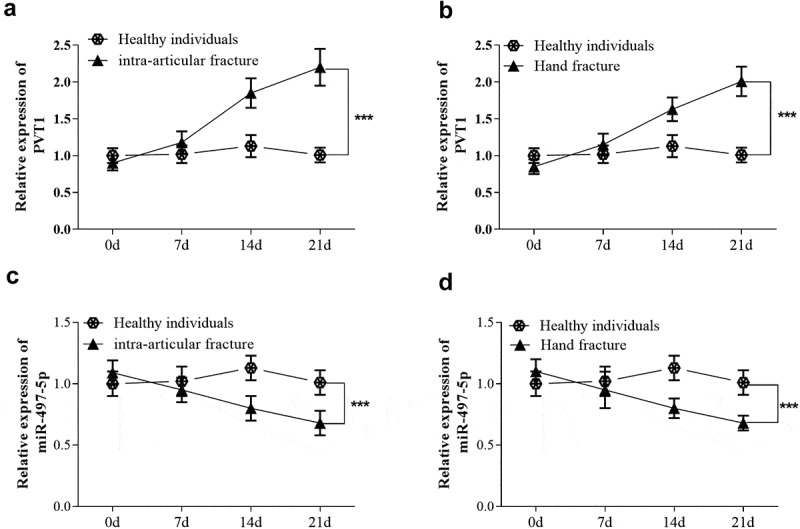
Serum samples were also collected from 80 fragility fracture patients at 7, 14, 21 days after standardized fixation therapy. Levels of lncRNA PVT1 (a) and miR-497-5p (b) were detected at different time points. *** P < 0.001, compared with healthy individuals

As shown in Figure 1a, qRT-PCR results showed no significant change in the level of lncRNA PVT1 in intra-articular fracture patients at 7 days after treatment compared with the control group. But as time went on, the level of PVT1 was significantly up-regulated at 21 days after treatment (*P* < 0.001). In hand fracture patients, similar expression changes of PVT1 levels were also found (Figure 1b). Besides, the expression level of miR-497-5p was opposite to that of PVT1 in the serum of patients. As shown in Figure 1c-d, the level of miR-497-5p showed a downward trend over time in both intra-articular and hand fracture patients, and the changes reached a significant level at 21 days after treatment (*P* < 0.001).

### PVT1 knockdown promoted cell proliferation and inhibited cell apoptosis in HFOB1.19 cells

**Figure 2.**
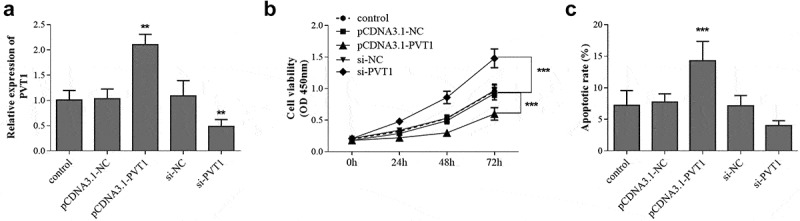
hFOB1.19 cells were transfected with pcDNA3.1-PVT1 or si-PVT1 to regulate gene expression (a). The cell viability (b) and apoptosis (c) were detected cell groups under different treatments. ** *P* < 0.01, *** *P* < 0.001, compared with control group

Firstly, the expression level of PVT1 in HFOB1.19 cells was regulated by cell transfection, and the transfection results were shown in Figure 2a. The cell viability test results of each group showed that overexpression of PVT1 significantly suppressed the proliferation of osteoblasts. On the contrary, cell viability was promoted after PVT1 knockdown (Figure 2b). The cell apoptosis rate showed an opposite trend. As shown in Figure 2c, the cell apoptosis was promoted by PVT1 overexpression, whereas it was inhibited by PVT1 knockdown.

### Targeted regulatory relationship between PVT1 and miR-497-5p

**Figure 3.**
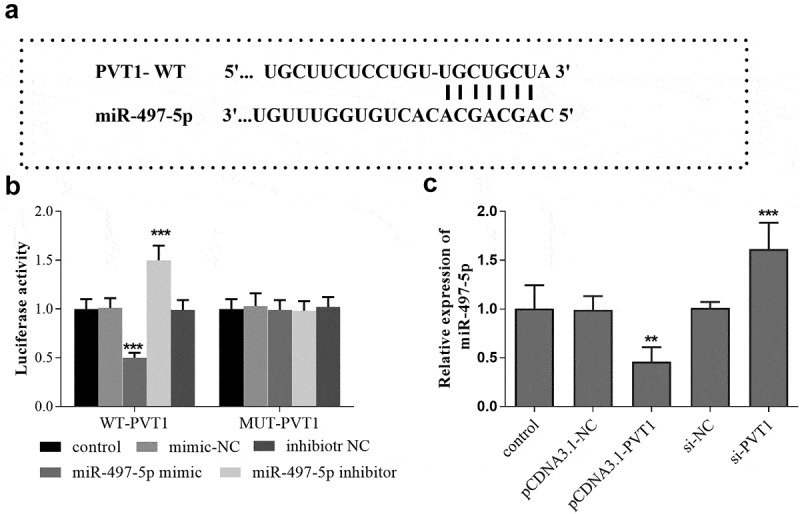
Targeted regulatory relationship between PVT1 and miR-497-5p. Starbase predicted the binding sites between PVT1 3ʹUTR and miR-497-5p (a). Luciferase activity assay was performed, and the luciferase activity (b) of cells in the different groups was detected. The levels of miR-497-5p (c) in cells under different transfection were measured using qRT-PCR. ** *P* < 0.01, *** *P* < 0.001, compared with control group

The binding sites between PVT1 3ʹUTR and miR-497-5p were shown in Figure 3a. Furthermore, the luciferase report analysis showed that the luciferase activity was significantly decreased after transfection of miR-497-5p mimics in PVT1-WT group, but the activity was significantly elevated after miR-497-5p inhibitor transfection (*P* < 0.001, Figure 3b). In the PVT1 MUT group, no significant changes were observed in the luciferase activity after transfection with miR-497-5p mimic or inhibitor (*P* > 0.05, Figure 3b). In addition, according to the qRT-PCR results, a reduced level of miR-497-5p was detected in cells with high PVT1 levels, while miR-497-5p levels were upregulated in cells after PVT1 knockdown (Figure 3c).

### Reverse effect of miR-497-5p downregulation on PVT1 knockdown

**Figure 4.**
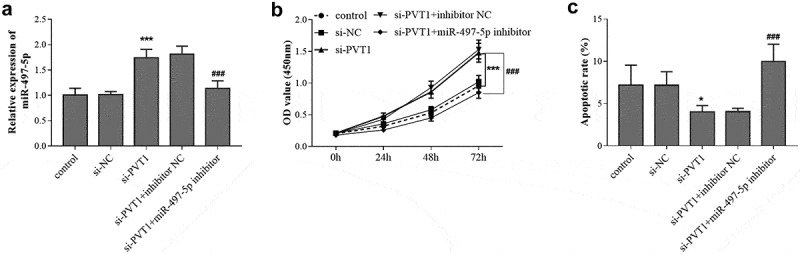
Reverse effect of miR-497-5p downregulation on PVT1 knockdown. Levels of miR-497-5p (a) were detected in cells under different transfection. The cell viability (b) and apoptosis (c) were detected cell groups under different treatment. * *P* < 0.05, *** *P* < 0.001, compared with control group; ^###^ *P* < 0.001, compared with si-PVT1 group

Considering the targeting relationship between PVT1 and miR-497-5p, the synergistic regulation of PVT1 and miR-497-5p on the behavior of osteoblasts were further explored. As presented in Figure 4a, PVT1 knockdown contributed to the increase of miR-497-5p level in HFOB1.19 cells, but the increased levels were abolished by miR-497-5p inhibitor transfection. The CCK-8 assay results demonstrated that si-PVT1 promoted the proliferation and inhibited the apoptosis of HFOB1.19 cells (Figure 4b-c). But the influence of *PVT1* knockdown on HFOB1.19 cell proliferation and apoptosis was reversed by miR-497-5p inhibition (Figure 4b-c).

### HMGA2 is the target gene of miR-497-5p

**Figure 5.**
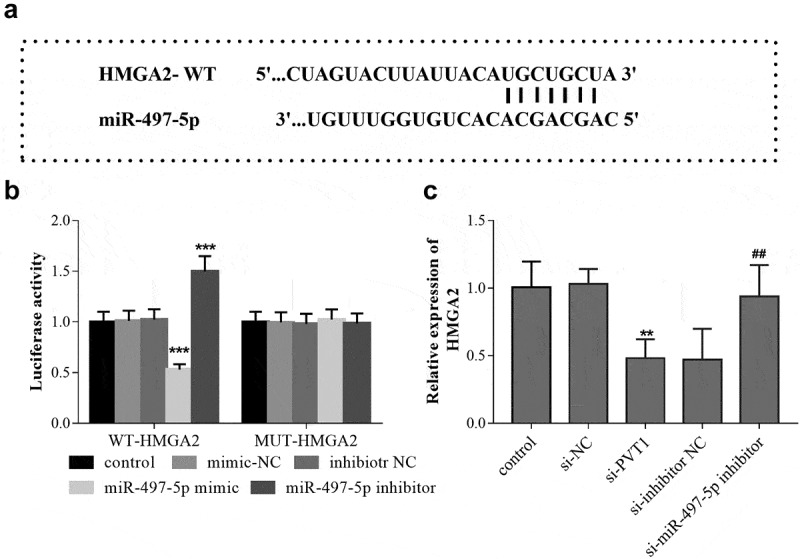
HMGA2 is the target gene of miR-497-5p. TargetScan predicted the binding sites between 3ʹ-UTR of HMGA2 and miR-497-5p (a). Luciferase activity assay was performed, and the luciferase activity (b) of cells in the different groups was detected. The levels of HMGA2 mRNA (c) in cells under different transfection were measured using qRT-PCR. ** *P* < 0.01, *** *P* < 0.001, compared with control group; ^##^ *P* < 0.01, compared with si-PVT1 group

The TargetScan analysis predicted that there are binding sites between 3ʹ-UTR of HMGA2 and miR-497-5p (Figure 5a). The luciferase report experimental results showed that in cells transfected with WT-HMGA2, miR-497-5p overexpression led to the reduction of the luciferase activity, while miR-497-5p downregulation caused the significant increase of cell luciferase activity (Figure 5b). In cells transfected with MUT-HMGA2, the cell luciferase activity can not be influenced by miR-497-5p levels (). Based on the qRT-PCR results in HFOB1.19 cells, PVT1 knockdown led to the downregulation of HMGA2, but the mRNA level of HMGA2 was upregulated by miR-497-5p inhibition (Figure 5c).

## Discussion

It is well known that most RNA molecules in an organism are translated and processed into product proteins, which subsequently play an important role in various physiological metabolism. However, there are other non-coding RNAs (ncRNAs) that are not translated into proteins. Among the many types of non-coding RNAs, lncRNAs subclass are more than 200 bp in length, and their expression levels are closely related to the pathogenesis and progression of various diseases. Fragility fracture is a common and serious complication of osteoporosis, which brings great pain and economic burden to patients. In recent years, a large number of studies have reported that abnormal expression of lncRNAs shows a close association with orthopedic diseases, and is involved in bone metabolism. For example, in fractured femoral neck tissues, elevated HAGLR levels are detected, which can promote the HFOB1.19 cell viability and accelerate the healing process of femoral neck fracture [23]. The abnormal expression of lncRNA KCNQ1OT1 in fracture healing has also been proved, which can affect the proliferation, migration and apoptosis of osteoblasts [24]. These findings all indicate the important role of lncRNAs in the process of fracture healing.

LncRNA PVT1 is located on human chromosome 8 and is homologous to the LncRNA PVT1 on mouse chromosome 15 [25]. The copy number of PVT1 is demonstrated to be significantly increased in patients with multiple cancers, such as renal cell carcinoma (RCC), lung cancer, esophageal cancer, and so on [26–28]. Recent studies have found that the expression level of PVT1 in chondrocytes of knee osteoarthritis is three times more than that of normal chondrocytes [11], and lncRNA PVT1 can regulate the apoptosis of chondrocytes of osteoarthritis through the adsorption of miR-488-3p, thus affecting the occurrence and development of osteoarthritis [12]. These findings reflect the crucial role of PVT1 in bone metabolism. In the current study, serum PVT1 levels were found to increase gradually over time after treatment for the fracture patients. It is known that the healing of fractures requires the proliferation of osteoblasts [29]. Considering the remarkable changes of serum PVT1 levels during fracture healing, its role in osteoblasts proliferation and apoptosis was further explored. The gain and loss function experiment results indicated that PVT1 silencing promoted cell proliferation and inhibited cell apoptosis in HFOB1.19 cells. Consistently, low levels of PVT1 were also detected in the serum of patients at the moment of fracture, which may be beneficial to the proliferation of osteoblasts, to meet the demand for a large number of osteoblasts in the early stage of fracture healing. Subsequently, the level of PVT1 gradually decreased, accompanied by the decrease of the proliferation rate of osteoblasts and the occurrence of further differentiation.

The competing endogenous RNAs (cRNAs) hypothesis mentions that lncRNAs competitively bind miRNAs through their miRNA response elements (mREs), then further regulating the expression of related genes [30]. In the present study, the level of miR-497-5p showed a downward trend over time in both intra-articular and hand fracture patients and the changes reached a significant level at 21 days after treatment, which was in contrast to the serum PVT1 levels. In addition, bioinformatics prediction and dual-luciferase reporter gene assay revealed a direct interaction between lncRNA PVT1 and miR-497-5p. Furthermore, the results of intracellular co-regulation showed that the influence of PVT1 knockdown on HFOB1.19 cell proliferation and apoptosis was reversed by miR-497-5p inhibition. MiR-497-5p has been suggested to participate in bone metabolism and is associated with progressive bone loss due to osteoporosis [31]. In osteoporosis patients, miR-497-5p is poorly expressed, and during HFOB1.19 cell differentiation, the expression of miR-497-5p was decreased [20]. Notably, miR-497-5p regulates several disease progression acting as the target gene of lncRNA PVT1 [21]. Based on the above findings, we concluded that PVT1 silencing might promote fracture healing via sponging miR-497-5p.

The effects of miRNAs on intracellular signal transduction and even the whole biological process vary with their complementary downstream target genes. In this study, we learned that HMGA2 was the downstream target gene of miR-497-5p in HFOB1.19 cells through dual-luciferase reporter gene assay. A lot of research shows that HMGA2 is involved in the regulation of proliferation and apoptosis of osteoblasts [32]. Consistently, Zhao et al. have reported that overexpression HMGA2 inhibited osteogenesis during the development of osteoporosis [32]. Another study reported by Zhang et al. also presents that HMGA2 is involved in the differentiation of human bone marrow-derived mesenchymal stem cells, which can differentiate into osteoblasts [33]. Therefore, we speculated that the promoting effect of miR-497-5p on the proliferation of osteoblasts and the inhibitory effect on the osteoblasts apoptosis might be partially completed through the down-regulation of HMGA2. Considering the important role of osteoblasts in fracture healing, we believe that miR-497-5p might promote fracture healing by targeting HMGA2.

## Conlcusion

In conclusion, the present study explored the novel function of lncRNA PVT1 silencing to accelerate fracture healing, and its possible molecular mechanism in terms of the regulation of the downstream miR-497-5p /HMGA2 axis. However, the interaction between miR-497-5p and HMGA2 and the details of the interaction need to be further explored. These conclusions may provide a new idea for the treatment of fragility fracture.

## Highlights

1. PVT1 was overexpressed gradually in fracture patients after treatment.
2. PVT1 knockdown promoted osteoblast proliferation and inhibited cell apoptosis.
3. PVT1 silencing may enhance fracture healing via mediating miR-497-5p/HMGA2 axis.
